# Influence of Genetic Polymorphisms on Response to Biologics in Moderate-to-Severe Psoriasis

**DOI:** 10.3390/jpm11040293

**Published:** 2021-04-12

**Authors:** Cristina Membrive Jiménez, Cristina Pérez Ramírez, Almudena Sánchez Martín, Sayleth Vieira Maroun, Salvador Antonio Arias Santiago, María del Carmen Ramírez Tortosa, Alberto Jiménez Morales

**Affiliations:** 1 Pharmacogenetics Unit, Pharmacy Service, University Hospital Virgen de las Nieves, 18014 Granada, Spain; cristina.membrive95@gmail.com (C.M.J.); almuweb06@gmail.com (A.S.M.); saylethvieira@gmail.com (S.V.M.); alberto.jimenez.morales.sspa@juntadeandalucia.es (A.J.M.); 2 Department of Biochemistry, Faculty of Pharmacy, Campus Universitario de Cartuja, University of Granada, 18071 Granada, Spain; mramirez@ugr.es; 3 Dermatology Service, University Hospital Virgen de las Nieves, 18014 Granada, Spain; salvadorarias@ugr.es

**Keywords:** psoriasis, pharmacogenetics, biological therapies, polymorphisms, response, biomarkers, personalized medicine, adalimumab, etanercept, ustekinumab

## Abstract

Psoriasis is a chronic inflammatory skin pathology of autoimmune origin and unknown etiology. There are various therapies for treating it, including a wide range of biopharmaceuticals indicated in moderate-to-severe psoriasis. Depending on their therapeutic target, they are classified as tumor necrosis factor inhibitors (anti-TNF) or cytokine inhibitors (interleukin-12, 23, and 17 antagonists). Although they have proved effective and safe, in clinical practice, many patients show a short- and long-term suboptimal response and even varying degrees of toxicity. This variability in response may be influenced by genetic factors, such as polymorphisms in the genes involved in the pathological environment, metabolism or mechanism of action of the drug that could affect the effectiveness and toxicity of biological therapies. This review assesses pharmacogenetic studies of the impact of genetic factors on response to biopharmaceuticals and toxicity in patients diagnosed with moderate-to-severe psoriasis. The results suggest that polymorphisms detected in the HLA genes, in genes that encode cytokines (*TNF*, IL genes, *TNFAIP3*), transporters (*PDE3A-SLCO1C1*, *SLC12A8)*, receptors (*TNFRSF1B*, *CD84*, *FCGR2A* and *FCGR3A*, *IL17RA*, *IL23R*, TLR genes, *PGLYRP4*) and associated proteins (*TNFAIP3*, *LY96*, *TIRAP*, *FBXL19*), as well as other genes implicated in the pathogenesis of psoriasis (*CDKAL1*, *CARD14*, *PTTG1*, *MAP3K1*, *ZNF816A*, *GBP6*, *CTNNA2*, *HTR2A*, *CTLA4*, *TAP1*) can be used in the future as predictive markers of treatment response and/or toxicity with biological therapies in patients diagnosed with moderate-to-severe psoriasis, tailoring treatment to the individual patient.

## 1. Introduction

Psoriasis is a chronic and recurrent inflammatory autoimmune skin disease with a worldwide prevalence of up to 8.5% in adults and 2.1% in children [1,2]. Apart from exceptional cases of erythrodermic or pustular psoriasis, the skin manifestations are not life-threatening. However, it severely affects quality of life, with a similar impact to diabetes or chronic obstructive pulmonary disease [3]. Furthermore, it is associated with other pathologies, such as erectile dysfunction in 35% of patients and potentially incapacitating arthropathy in 40% [4,5,6]. In short, psoriasis is regarded as a systemic entity rather than an exclusively dermatological disease [7].

**Figure 1:**
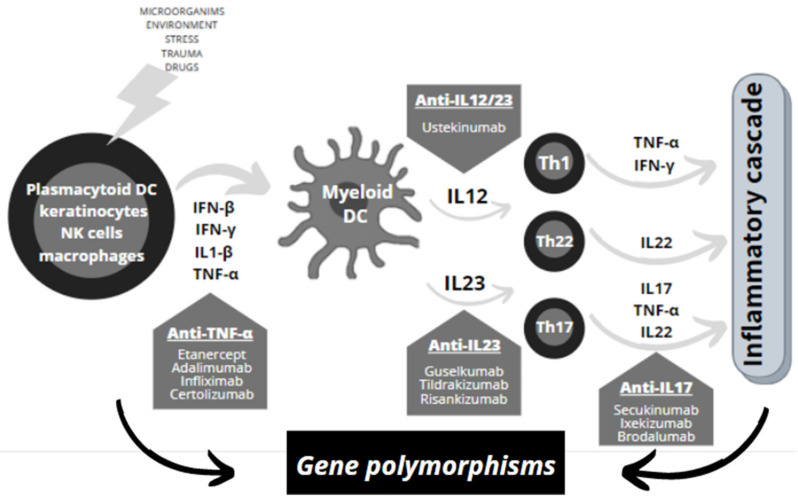
Summary of the main cells and inflammatory cytokines involved in developing psoriasis and the role of gene polymorphisms in biopharmaceuticals. Combination of genetic, immunological and environmental factors (such as stress, trauma, medications and microorganism infections, among others) lead to activation of plasmacytoid dendritic cells, keratinocytes, natural killer cells and macrophages that secrete cytokines (IFN-β and IFN-γ, IL1B, TNF). Activated dendritic cells promote the production of IL-12 and IL-23, which regulate the differentiation and proliferation of helper T lymphocytes (Th1, Th17 and Th22), which produce more cytokines (TNF, IFN-γ, IL22, IL-17). This inflammatory cascade produces a hyperproliferation of keratinocytes in the epidermis and in the vascular endothelium, giving rise to epidermal hyperplasia and psoriasis development. Targeted therapy with monoclonal antibodies inhibits different cytokines of this pathway (TNF, IL12/23, IL17, IL23), preventing the development of the inflammatory cascade and subsequently epidermal hyperplasia, typical of psoriasis. Genetic variations, such as single nucleotide polymorphisms in genes encoding these cytokines, receptors or proteins involved in this mechanism, can be associated with response or toxicity to treatment with biologic therapies. Plasmacytoid DC: plasmacytoid dendritic cells; NK cells: natural killer T cells; Myeloid DC: myeloid dendritic cells; Th: T helper lymphocytes; IFN: interferon; IL: interleukin; TNF: tumor necrosis factor; Anti-TNF: TNF inhibitor drugs; Anti-IL drugs: cytokine inhibitor drugs.

Its etiology is unclear, although it is thought that it could be due to a combination of genetic, immunological and environmental factors (such as stress, trauma, medications and microorganism infections, among others) (Figure 1) [8]. It has been found that the incidence differs between ethnicities and that it is greater among relatives and even more between monozygotic twins [9]. In addition, genetic variants in molecules that influence developing epidermal hyperplasia led to an increased susceptibility to develop this pathology. In particular, the HLA-Cw*06 alleles (also known as HLA-C*06:02) have been described as the first-factor risk of psoriasis. Remarkably, HLA-Cw*06 was found to mediate autoimmunity against melanocytes through the ability of its protein product to present ADAMTS-like protein 5 (ADAMTSL5). In fact, this complex is recognized by epidermal CD8+ T cells, which directly target melanocytes and produce inflammatory cytokines (such as TNF-α and IL-17) that, in turn, are able to alter melanocyte functions and proliferation, leading to dysregulation of skin homeostasis [10].

Abnormalities in cutaneous immune responses, both innate and adaptive, are responsible for the development and maintenance of psoriatic inflammation [8,11]. One of the main pathogenic mechanisms is based on the activation of plasmacytoid dendritic cells, keratinocytes, natural killer cells and macrophages that secrete cytokines, such as beta and gamma interferons (IFN-β and IFN-γ), interleukin 1 beta (IL1B) and tumor necrosis factor (TNF), leading to the activation of myeloid dendritic cells by Toll-like receptors (TLRs) [12]. Activated dendritic cells promote the production of IL-12 and IL-23, which regulate the differentiation and proliferation of helper T lymphocytes (Th1, Th17 and Th22). Th1 cells produce TNF and IFN-γ, Th2 cells produce IL22, while Th17 cells, as well as producing TNF and IL-22, also secrete IL-17. These cytokines activate the proliferation of keratinocytes in the epidermis, and this inflammatory cascade produces a hyperproliferation of keratinocytes in the epidermis and in the vascular endothelium, giving rise to epidermal hyperplasia and psoriasis development (Figure 1) [13]. Furthermore, the status of keratins and collagen has been linked to the development of keratinocyte hyperproliferation, typical of psoriatic lesions. Keratins are involved in cell proliferation and inflammatory response by mediating interactions between cells or cells and the extracellular matrix, while collagen supports stabilization of tissue, strength and resilience. Currently, 54 keratin genes (KRTs) have been identified, and six genetic variations that consequently produce abnormal keratinocytes in the psoriatic epidermis (*KRT1*, *RT6A*, *KRT6B*, *KRT10*, *KRT16* and *KRT17).* Moreover, altered collagen structure and function due to relevant mutations can affect all tissues or organs and cause various pathological phenotypes. Specifically, two genetic variants of the collagen (*COL*) genes, *COL8A1* and *COL8A2,* have been shown to be associated with the extracellular matrix remodeling and angiogenesis that occurs in psoriasis. In addition, the impact of genetic variants in *COL* may be associated with the development of psoriatic arthritis in patients with cutaneous psoriasis [10].

Finally, the relationship of the human microbiome with the immune system and the skin barrier should be highlighted. Analysis of the microbiota of psoriatic lesions revealed the presence of bacteria (*Staphylococcus aureus*, *Streptococcus pyogenes*), viruses (*Human papillomavirus*) and fungi (*Candida Albicans),* suggesting that developing this pathology may be related to an excessive immune response to microbial pathogens. It has been observed that *C. Albicans* could even exacerbate skin dysfunction by disrupting the physiological activity of immune-related factors, including TLR [10].

Therefore, the co-occurrence of genetic, epigenetic and non-genetic factors could explain specific skin phenotypes and the differential susceptibility to psoriasis [10].

Ninety percent of patients develop clinical manifestations in the form of erythematous plaques covered by whitish scales on the scalp, elbows, knees and back [14,15,16]. The severity of the lesions is measured with the psoriasis area severity index (PASI), body surface area (BSA) and dermatology life quality index (DLQI) indicators. The latest consensus document on the evaluation and treatment of psoriasis established that moderate-to-severe psoriasis is psoriasis with PASI > 10, BSA > 10 and DLQI > 10 [17]. In addition, the effectiveness of treatment is evaluated by absolute PASI or percentage improvement in PASI; for example, a 90% reduction (PASI 90) [1].

The treatments used are aimed at blocking the inflammatory response. As an exceptional non-pharmacological treatment, exposure to sunlight is recommended [18]. However, pharmacological treatment depends on the severity of psoriasis, and topicals, phototherapy, traditional oral immunomodulators, or biological therapy may be used [19]. In mild psoriasis, the treatment is based mainly on topical and symptomatic therapy (corticosteroids, calcineurin inhibitors, topical retinoids vitamin D analogs) [20]. When the disease is more severe (BSA > 10, PASI > 10 and DLQI > 10), systemic therapy (methotrexate, cyclosporine, acitretin, apremilast, fumaric acid esters), phototherapy (UV, UVB, PUVA) or photochemotherapy [therapy based on psoralens plus ultraviolet A radiation (PUVA)] are recommended. As a last option, when the severity indicators are greater than 10, and there is no response to previous treatments or contraindicated, treatment with biologics is used [17].

There is a wide range of biopharmaceuticals indicated in moderate-to-severe psoriasis [21]. They are classified into two groups according to the therapeutic target: tumor necrosis factor inhibitor (anti-TNF) therapies, such as infliximab (INF), etanercept (ETN), adalimumab (ADA) and certolizumab (CTL), and cytokine inhibitors: ustekinumab (UTK), secukinumab (SCK), ixekizumab (IXE), brodalumab (BDL), guselkumab (GSL), tildrakizumab (TDK), the recently approved risankizumab (RSK) and another two new drugs that are undergoing trials (bimekizumab and mirikizumab).

The first treatment options for moderate-to-severe plaque psoriasis are the anti-TNF drugs ADA and ETN and the IL-12/23 inhibitor UTK [22,23]. Second-line treatments include the IL-17 inhibitors SCK and IXE and BDL targeting the IL-17RA receptor [24,25]. There are also GSL, TDK and RSK, which inhibit IL-23 or its receptor IL-23R (Figure 1) [26,27].

Anti-TNFs were the first biologics indicated in psoriasis to be marketed. A network meta-analysis has indicated that INF is the most effective (80% of patients reached PASI 75 at week 10), followed by CTL and ADA, with similar degrees of efficacy (*n* early 80% of patients reached PASI 75 at week 16), and finally ETN (49% of patients reached PASI 75 at week 12) [28]. In addition, four recent meta-analyses have evaluated the short-term efficacy of all the biological therapies (anti-TNFs and cytokine inhibitors), and the two with the largest numbers of patients (77 studies/34816 patients and 28 studies/9940 patients) showed that the most effective drugs (PASI 90 at 12–16 weeks of treatment) are RSK (80.9%), BDL (76.8%), IXE (71.6%) and GSL (76.8%), followed by SCK (66%), INF (56%), UTK (58–43.6%, depending on dosage) and finally the anti-TNFs ADA (44.2%), CTL (38.8–43.7%, depending on dosage) and ETN (16.8–26.3%, depending on dosage) [24,26,29,30]. These results were confirmed in head-to-head clinical trials, demonstrating that the IL-17 or IL-23 inhibitor drugs are more effective than the IL-12/IL-23 inhibitors and anti-TNFs [31]. However, BDL, IXE and SCK have the highest probability of maintaining long-term efficacy (40–64 weeks) (97, 83 and 77%, respectively) [32].

As regards safety, all of them have proved to be very safe; patients show no increase in rates of severe infections or internal malignancies [33]. It should be noted that UTK and SCK have the lowest rates of adverse effects (compared to other anti-TNF biological therapies), even in patients with comorbidities, in the case of SCK [34,35].

Despite the confirmed efficacy and safety of these medications, not all patients obtain good results. Some do not show the expected response in the induction phase (16–24 weeks with the treatment) or undergo a loss of response in the maintenance phase (from 24 weeks to several years) [36]. Moreover, certain patients experience various degrees of toxicity. This variability in short- and long-term response, as well as toxicity, may be due to genetic factors. Therefore, this genetics variant can be used in the future as predictive markers of treatment response and/or toxicity with biological therapies in patients diagnosed with moderate-to-severe psoriasis, tailoring treatment to individual patients.

In light of all the foregoing, the object of this review of pharmacogenetic studies of candidate genes is to assess the impact of genetic variants on the response to treatment with biopharmaceuticals in patients diagnosed with moderate-to-severe psoriasis.

## 2. Materials and Methods

**Figure 2:**
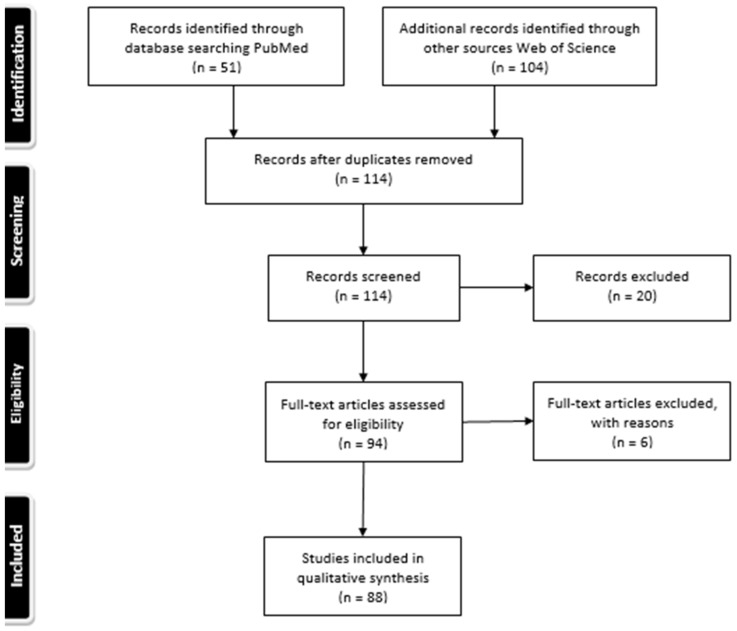
Flow chart regarding study selection.

A PubMed search included key words “psoriasis”, “psoriatic”, together with “treatment”, “biological therapy”, “etanercept”, “infliximab”, “adalimumab”, “ustekinumab”, “secukinumab”, “certolizumab pegol”, “guselkumab”, “tildrakizumab”, “brodalumab”, “ixekizumab”, “risankizumab” and “polymorphisms” and “response” or “toxicity”. Data regarding gene, SNP, year of publication, number of patients, population, drugs, response (time and outcome measure), results (odds ratio, 95% confidence interval and *p*-value), and allele or genotype of response were recorded. Figure 2 shows a flow chart regarding study selection.

## 3. Pharmacogenetics of Biological Therapies in Psoriasis

Alterations in the genes involved in the pathological environment of the disease, metabolism or mechanism of action may influence the effectiveness of biopharmaceuticals in psoriasis. Specifically, genetic polymorphisms in the human leukocyte antigens, cytokines, receptors, transporters and associated proteins, as well as other genes implicated in the physiopathogenesis of the disease, have been shown to play a crucial role in interindividual variability in response to these drugs (Table 1, Table 2 and Table 3).

### 3.1. Human Leukocyte Antigens (HLAs)

The human leukocyte antigens (HLAs) are part of the major histocompatibility complex (MHC) and help to identify exogenous proteins that may trigger an immune response. The HLA system is located at the *PSORS1* locus of chromosome 6 and encodes a large number of HLAs with different functions [37]. They are grouped into three classes: class I (A, B and C) is responsible for presenting peptides of intracellular origin, while class II presents antigens to T cells, and class III encodes enzymes, some complement proteins, and other proteins that interfere in antigen presentation, but are not HLAs [38].

Many variant alleles of HLA-I and HLA-II genes have been described, and their association with the risk of developing psoriasis or relationship to response to biological therapy (anti-TNF and UTK) have been studied (Table 1). The *HLA-A* rs9260313 (T > A, C) polymorphism has been studied in a cohort of Spanish patients (*n* = 109), finding an association between the GT haplotype (*TRAF3IP2* rs13190932 and *HLA-A* rs9260313, respectively) and response (PASI 75 at eight months) to INF and ADA, but not to ETN (*p* < 0.005) [39]. However, this association was not statistically significant when the *TRAF3IP2* rs13190932 and *HLA-A* rs9260313 polymorphisms were studied separately.

In addition, several studies have evaluated the usefulness of *HLA-B* as a predictor of response to biologics in patients diagnosed with moderate-to-severe psoriasis [40,41]. A study with Spanish patients (*n* = 81) found that the *HLA-B*/*MICA* rs13437088 polymorphism was related to response to ETN at 3 months (OR = 589.99, CI_95%_ = 2.71–128,614.40, *p* = 0.02) [40]. Another study assessed the impact of the *HLA-B* haplotype (HLA-B*46) in Asian patients (from China) treated with ETN or UTK (*n* = 74), without obtaining statistically significant results (ETN: *n* = 45, *p* = 1.0; UTK: *n* = 29, *p* = 0.32) [41].

The *HLA-C* haplotype has been extensively studied in psoriasis. In particular, the rs12191877 polymorphism was associated with response to anti-TNF medication (PASI 75) at 3 months in 144 Spanish patients (*HLA-C* rs12191877-T: OR = 0.30, CI_95%_ = 0.11–0.88, *p* = 0.027) [42]. Similarly, a study performed in Greek patients (*n* = 250) showed that the rs1048554 polymorphism, situated close to the *HLA-C* locus, was associated with better response to anti-TNF drugs (PASI 75 at 6 months: OR = 3.94, CI_95%_ = 1.16–13.3, *p* = 0.0032), specifically with ADA (*p* = 0.0007) [43]. Moreover, patients carrying the rs610604-A allele (situated at the HLA locus) showed a better response to ADA (*p* = 0.05) [43].

The HLA-Cw*06 alleles (also known as HLA-C*06:02) have been shown to confer a high risk of suffering from psoriasis, but its association with response to anti-TNF drugs and cytokine inhibitors is contradictory [20,39,44,45,46]. A study in Spanish patients (*n* = 109) observed that patients carrying the HLA-Cw*06 allele had a lower probability of responding (PASI 75 at week 24) to ADA, ETN or INF (OR = 58.1, CI_95%_ = 71.7–93.8, *p* = 0.049) [47]. In addition, a study with 116 Spanish patients showed that those are carrying the HLA-Cw*06 alleles together with the deletion of the two late cornified envelope genes (*LCE3C_LCE3B-del*), which has been evaluated independently without statistically significant results, had a higher probability of responding to treatment with anti-TNF drugs (OR = 3.14, CI_95%_ = 1.07–9.24, *p* = 0.034) [48,49,50,51]. However, it was not possible to predict the response to anti-TNF medication in a study with patients from the United Kingdom and Ireland (*n* = 138) treated with ADA or ETN [45]. In a Italian population the HLA-Cw*06 allele was not associated with response to ADA or ETN (*n* = 123 and *n* = 96; PASI 75 at 3 months; *p* > 0.05) [44,52]. Similarly, in a Italian population, Talamonti et al. found no association between the HLA-Cw*06 allele and response to anti-TNF medication (*n* = 122), but in patients treated with UTK (*n* = 51) they observed that the HLA-Cw*06 allele could be a predictor of good response (PASI 75 or PASI 90 at week 12) (PASI 75: OR = 13.4, CI_95%_ = 1.6–12.6, *p* < 0.008; PASI 90: OR = 4.6, *p* = 0.02), rapid response (PASI 75 at week 4) (OR = 5.36, CI_95%_ = 1.24–23.1, *p* = 0.024) and lasting response (PASI 75 or PASI90 at week 40) (PASI 75: OR = 3.9, CI_95%_ = 2–7.37, *p* = 0.014; PASI 90: OR = 8.7, *p* = 0.012) to UTK [49,53].

These results were confirmed in two larger cohorts of patients treated with UTK (*n* = 134; PASI 75 at week 12 (OR = 4.1, *p* = 0.001) and week 52 (OR = 3.7, *p* = 0.003), and *n* = 255; PASI 75 at week 12 (OR = 3.28, CI_95%_ = 1.92–5.59, *p* < 0.001) and week 52 (OR = 3.82, CI_95%_ = 1.88–7.73, *p* < 0.001)) [54,55]. They were also replicated in Asian patients from China (*n* = 66; PASI 75 at week 16: OR = 0.28, CI_95%_ = 0.11–0.68, *p* = 0.005) and in patients of North American (United States) origin (*n* = 523; PASI 50 at week 4; 92.8% vs. 80.5%; PASI 75 at week 12:82.6% vs. 64.4%), confirming the results obtained previously [56,57]. Moreover, a study with Italian population treated with UTK (*n* = 64) showed that HLA-Cw*06 can be a predictor of treatment response [58]. The presence of the *IL12B* rs3212227-C, *IL12B* rs6887695-G and *IL6* rs1800795-C alleles was associated with response to UTK in patients carrying the HLA-Cw*06 allele [*HLA-Cw6* + *L12B* rs3212227-C: PASI 75 at week 4 (OR = 10.49, CI_95%_ = 50–0, *p* = 0.009) and at week 52 (OR = 5.21, CI_95%_ = 83.3–44.4, *p* = 0.007); *HLA-Cw6* + *IL12B* rs6887695-GG: PASI 75 at week 4 (OR = 6.11, CI_95%_ = 23.5–10.5, *p* = 0.031) and at week 52 (OR = 4.75, CI_95%_ = 82.4–42.1, *p* = 0.006); *HLA-Cw6* + *IL6* rs1800795-C: PASI 75 at week 4 (OR = 6.52, CI_95%_ = 38.5–0, *p* = 0.027) and at week 52 (OR = 4.99, CI_95%_ = 84.9–46.7, *p* = 0.005) [58]. These results were confirmed by a subsequent meta-analysis, which evaluated 8 studies with 1048 Caucasian and Asian patients diagnosed with moderate-to-severe psoriasis and treated with UTK (HLA-Cw*06 (+): PASI 75 at six months; OR = 0.24, CI_95%_ = 0.14–0.35, *p* < 0.001] [46]. However, a recent study looking for predictive biomarkers of biological treatment response in psoriasis in a population (United Kingdom) (*n* = 1326) found that the maximum response to ADA treatment was reached at 6 months in patients, who did not carry the HLA-Cw*06 allele and had developed psoriatic arthritis (PASI 90 at 6 months: OR = 2.95, *p* = 5.85 × 10^–7^) [59].

Finally, concerning *HLA-G*, the only association to have been studied is that of the *HLA-G* 14-pb Ins/Del polymorphism (rs66554220) with response to anti-TNF treatment in a preliminary study with the Italian population (*n* = 11), without finding statistically significant results [60] (Table 1).

### 3.2. Cytokines and Associated Proteins

#### 3.2.1. Tumor Necrosis Factor (TNF)

The tumor necrosis factor (*TNF*) gene, formerly known as *TNF-α* or *TNFA*, belongs to the TNF superfamily and is located on chromosome 6 (6p21.33). This gene encodes a proinflammatory cytokine with multiple functions, including inducing the production of T-cells, which trigger the local proliferation of epidermal keratinocytes. It, therefore, plays an essential role in the pathogenesis of psoriatic lesions [61,62]. Moreover, TNF is the target of three biopharmaceuticals (ADA, INF and CTL) indicated in the treatment of moderate-to-severe psoriasis [63]. Therefore, alterations in the *TNF* gene are directly related to the efficacy of these drugs (Table 2).

More than 200 variants of this gene have been found, four of which are the most extensively studied in psoriasis. The *TNF*-308 rs1800629 (A > G), *TNF*-238 rs361525 (G > A), and *TNF*-857 rs1799724 (C > T) polymorphisms have been associated with the risk of suffering from psoriasis and with response to anti-TNF drugs in autoimmune pathologies, mainly ankylosing spondylitis, inflammatory bowel disease, psoriatic arthritis and psoriasis [20,46,64]. The impact of these variants in autoimmune diseases has been evaluated in a meta-analysis that included 13 studies with 887 Caucasian and Asian patients treated with ETN, ADA and INF [64]. The *TNF*-308 rs1800629 (A > G) polymorphism was assessed in 11 studies (807 patients), showing an association between the G allele and the response to anti-TNF drugs in Caucasian patients (OR = 2.005, CI_95%_ = 1.417–2.838, *p* = 0.000086) [64,65]. However, this association was not statistically significant in Asian patients (2 studies/140 patients). Similarly, the *TNF*-238 rs361525-G allele showed an association with anti-TNF medication in Caucasian patients (4 studies/500 patients) (OR = 2.196, CI_95%_ = 1161–4.154, *p* = 0.016) that was likewise not confirmed in Asian patients (1 study/100 patients) [64]. In line with these results, a study performed in Spanish patients diagnosed with moderate-to-severe psoriasis (*n* = 109) showed that patients with the *TNF*-238 rs361525-G allele showed a better response to anti-TNF treatment (PASI 75 at six months) (*p* = 0.049) [47]. The impact of the *TNF*-857 rs1799724 (C > T) polymorphism on the response to anti-TNF drugs was evaluated in Caucasian (4 studies/483 patients) and Asian patients (1 study/100 patients); Caucasian patients carrying the *TNF*-857 rs1799724-C allele showed a better response (OR = 1.779, CI_95%_ = 1.130–2.802, *p* = 0.013), which was not confirmed in Asian patients [64,66]. Specifically, a meta-analysis comprising 2 studies and 177 Caucasian patients with psoriatic disease treated with anti-TNF medication found that patients carrying the *TNF*-857 rs1799724-C allele responded better to treatment with ETN (OR = 2.238, CI_95%_ = 1.319–3.798, *p* = 0.003) [64,67,68]. Similar results were also obtained in a study with 109 Spanish patients diagnosed with moderate-to-severe psoriasis and treated with anti-TNF drugs; *TNF*-857 rs1799724-C patients had better BSA (83.1% vs. 92.7%, *p* = 0.004) and PASI (82.7% vs. 92.6%, *p* = 0.009) scores and better response at 6 months (PASI 75:71.4% vs. 96.3%, *p* = 0.006) [47].

Finally, in the promoter region of the TNF gene is the *TNF*-1031 rs1799964 (T > C) polymorphism. A study with 109 patients of Spain diagnosed with psoriasis showed an association between patients carrying the TT genotype of the *TNF*-1031 polymorphism and anti-TNF response at 3 and 6 months (3-month PASI 75:90.8% vs. 75.7% (*p* = 0.047); 6 month PASI 75:85.5% vs. 65.7% (*p* = 0.038)); specifically, INF achieved the highest response at 3 months (PASI 75:84.2% vs. 42.9%, *p* = 0.024; PASI 90:73. 7% vs. 28.6, *p* = 0.015) and at 6 months (PASI 75:94.1% vs. 53.8%; *p* = 0.025; PASI 90:76.5% vs. 30.8%, *p* = 0.025; ΔPASI: 94.1% vs. 64.7%, *p* = 0.019) [47].

#### 3.2.2. Interleukin 1 Beta (IL1B)

In the 2q14.1 region of chromosome 2 is the interleukin 1 beta gene (*IL1B* or *IL1F2*), which encodes a protein crucial to developing acute-phase response [69]. The IL1B cytokine induces prostaglandin synthesis, neutrophil influx and activation, T-cell activation and cytokine production, B-cell activation and antibody production, as well as promoting differentiation of T-helper 17 (Th17) cells and combining with IL-12 to induce IFN-γ synthesis from Th1 cells [70]. In short, IL1B is a very potent proinflammatory cytokine, and possible genetic alterations could greatly influence the response to anti-TNF drugs or cytokine inhibitors. There are two known genetic alterations in *IL1B*, rs1143623 (C > A/C > G) and rs1143627 (G > A), associated with response to anti-TNF treatment and to UTK [71].

A study with 478 patients from Denmark diagnosed with moderate-to-severe psoriasis and treated with anti-TNF medication (*n* = 376) and with UTK (*n* = 230) assessed the effect of these polymorphisms on the treatment response (PASI 75 at 3 months) [71]. Patients carrying the *IL1B* rs1143623-GG or *IL1B* rs1143627-AA genotypes showed worse response (*IL1B* rs1143623 treated with anti-TNF drugs (OR = 0.35, *p* = 0.0041, q = 0.19) and with UTK (OR = 0.25, *p* = 0.0049); *IL1B* rs1143627 treated with anti-TNF (OR = 0.28, *p* = 0.0016, q = 0.19) and with UTK (OR = 0.24, *p* = 0.0042, q = 0.19)) [71].

#### 3.2.3. Interleukin 6 (IL6)

Cytokine 6 (IL6), also known as interferon-β2, is a protein encoded in humans by the *IL6* gene located on chromosome 7 (7p15.3). This cytokine acts in the acute phase of inflammation and in B-cell maturation [72]. Therefore, genetic alterations in the *IL6* gene may give rise to a modification of the response to anti-TNF drugs.

The *IL6* rs1800795 (C > G,T) polymorphism has been studied in a small cohort of the Italian population treated with anti-TNF medication (*n* = 60) [73]. It was observed that obese patients carrying the *IL6* rs1800795-GG genotype had a worse response to anti-TNF drugs than patients carrying the CG and CC genotypes (OR = 2.00, CI_95%_ = 1.19–3.38, *p* ≤ 0.05) [73].

#### 3.2.4. Interleukin 12B (IL12B)

The interleukin 12B *(IL12B*) gene, also known as natural killer cell stimulatory factor 2, is located on chromosome 5 (5q33.3) [74]. This gene codes for the p40 subunit of IL12, which acts on T and natural killer cells; it is important for maintaining Th1 memory cells and associates in turn with IL23A to form interleukin 23 (IL-23). Interleukin 23 induces developing inflammation and may, therefore, be responsible for autoimmune inflammatory diseases [74]. Genetic alterations in *IL12B* may influence the response to biologics indicated for the treatment of moderate-to-severe psoriasis, especially UTK, an IL-12/23 inhibitor. Two clinically significant polymorphisms have been found: *IL12B* rs2546890 (A > G) and *IL12B* rs3213094 (C > G,T).

The *IL12B* rs2546890 polymorphism has been evaluated in two studies conducted simultaneously with Spanish patients treated with ETN (*n* = 78) or anti-TNF drugs (ADA, ETN, INF) (*n* = 144) [40,42]. Patients diagnosed with plaque psoriasis, who developed psoriatic arthritis treated with ETN for 6 months and, who carried the *IL12B* rs2546890-G allele showed worse response (PASI 75: OR = 11.92, CI_95%_ = 1.07–132.67, *p* = 0.044) [40]. Subsequently, it was confirmed that for patients carrying the *IL12B* rs2546890-G allele, anti-TNF drugs are less effective (PASI 75) at 3 months (OR = 3.22, CI_95%_ = 1.23–8.40, *p* = 0.017), at 6 months (OR = 4.14, CI_95%_ = 1.23–13.81, *p* = 0.022) and after a year of treatment (OR = 2.79, CI_95%_ = 1.02–7.64, *p* = 0.046), compared to patients with the *IL12B* rs2546890-A allele [42].

In addition, the *IL12B* rs3213094 polymorphism was studied in patients from the Netherlands treated with ETN, ADA and UTK (*n* = 234) and an association were found with the response to UTK (*n* = 66) (ΔPASI at week 12) [50]. Patients with the *IL12B* rs3213094-CT genotype showed a better response to UTK than those carrying the *IL12B* rs3213094-CC genotype (beta = −3.16, CI_95%_ = −5.72–−0.59, *p* = 0.017). However, no statistically significant results were found for ETN and ADA. [50].

#### 3.2.5. Interleukin 17 (IL17) Genes

The *IL17A* and *IL17F* genes, belonging to the interleukin 17 (IL17) family, are located on chromosome 6 (6p12.2) and encode the IL-17A and IL-17 F cytokines, which bind to the IL-17RA receptor [75]. Cytokine 17 performs an important role in the innate and adaptive immune system, activating and recruiting neutrophils as an antibacterial defense in areas of infection [76]. They have also been associated with autoimmune and inflammatory diseases, such as psoriasis since IL-17 values are increased in psoriasis lesions [77]. The impact of this gene on certain autoimmune and inflammatory diseases, such as psoriasis, has led to the development of two drugs aimed at blocking this cytokine (SCK and IXE) [78]. Recent studies show an association between polymorphisms of IL17 genes and response to anti-TNF medication and UTK [79,80,81].

A study in Spanish patients (*n* = 194) showed that the *IL17F* rs763780 polymorphism was a useful predictor of response to UTK (*n* = 67) and to the anti-TNF medications INF (*n* = 35) and ADA (*n* = 62) [81]. In particular, patients carrying the *IL17F* rs763780-CT genotype showed worse response to UTK (PASI 75 at week 16: OR = 12.23, CI_95%_ = 1.17–127.36, *p* = 0.022, and at week 28: OR = 14.18, CI_95%_ = 1.35–149.42, *p* = 0.016). This genotype (*IL17F* rs763780-CT) was also associated with worse response to ADA treatment (*n* = 62) (PASI 75 at week 28: OR = 14.00, CI_95%_ = 2.15–91.12, *p* = 0.0044). Conversely, in patients treated with INF (*n* = 35), the *IL17F* rs763780-CT genotype was associated with better treatment response (PASI 75 at week 16 (*p* = 0.023) and week 28 (*p* = 0.02)) [81].

#### 3.2.6. Tumor Necrosis Factor Alpha-Induced Protein 3 (TNFAIP3)

The tumor necrosis factor-alpha-induced protein 3 (*TNFAIP3*) gene is located in the 6q23.3 region [82]. The protein encoded by this gene inhibits NF-κβ activation, as well as TNF-mediated apoptosis, and has ubiquitin ligase and deubiquitinase activity involved in the cytokine-mediated immune response [83,84]. The genetic alterations of *TNFAIP3* have been extensively studied in various pathologies. In psoriasis, however, only the effect of the *TNFAIP3* rs610604 (G > T) and *TNFAIP3* rs6920220 (G > A) polymorphisms have been evaluated.

The *TNFAIP3* rs610604 polymorphism has been assessed in four studies, with inconclusive results. First, the influence of this polymorphism on the response to UTK (PASI 75 at 40 weeks) was studied in the Italian population diagnosed with moderate-to-severe psoriasis (*n* = 51), without finding any statistically significant association (OR = 1.6, *p* = 0.75) [49]. Similarly, it was not possible to associate the *TNFAIP3* rs610604 polymorphism with UTK response (PASI 75 at weeks 4, 12, 28, 40 and 52) in 64 Italian populations (*p* > 0.05) [58]. Subsequently, a study with 66 patients from the Netherlands diagnosed with psoriasis showed a significant association between the *TNFAIP3* rs610604 polymorphism and response to UTK (ΔPASI at week 12). In particular, patients carrying the GG genotype had a worse response to UTK (beta = 3.49, CI_95%_ = 0.33–6.65, *p* = 0.031). Moreover, the regression model adjusted to those patients who developed psoriatic arthritis showed an association between the polymorphism and treatment response (beta = 11.23, CI_95%_ = 0.48–7.07, *p* < 0.001). However, no significant association was found in patients treated with ADA or ETN (*n* = 282; *p* > 0.05) [50].

Recently, a preliminary study with 20 Spanish patients diagnosed with psoriasis and psoriatic arthritis assessed the relationship between these polymorphisms (*TNFAIP3* rs610604 and *TNFAIP3* rs6920220) and improvement in the quality of life of patients treated with anti-TNF drugs (European quality of life visual analog scale (EQ-VAS) score at 3 and 6 months), finding statistically significant results at three months of treatment (*TNFAIP3* rs610604-AC/CC: OR = −10.60, CI_95%_ = −20.71–−0.48, *p* = 0.041; *TNFAIP3* rs6920220-AA: OR = −25.83, CI_95%_ = −47.969–−3.698, *p* = 0.025) [85].

### 3.3. Transporters, Receptors and Associated Proteins

#### 3.3.1. Phosphodiesterase 3A (PDE3A)-Solute Carrier Organic Anion Transporter Family Member 1C1 (SLCO1C1)

The *PDE3A* and *SLCO1C1* genes are located on chromosome 12 (12p12.2). *PDE3A* is expressed mainly in cardiac tissue and codes for a phosphodiesterase responsible for internal control of nucleotide signaling, whereas *SLCO1C1* encodes a sodium-independent transporter with a high affinity for the thyroid hormones in brain tissue and is related to various pathologies [86,87].

The *SLCO1C1* rs3794271 (G > A, C) and *PDE3A* rs11045392 (T > A, C) polymorphisms are in linkage disequilibrium and have been evaluated in a study with 130 Spanish patients diagnosed with moderate-to-severe psoriasis and treated with anti-TNF medication (*n* = 130) [88]. This study demonstrated that patients carrying the *SLCO1C1* rs3794271-G allele obtained a better response to anti-TNF drugs (ΔPASI at 3 months) (*p* = 0.00057) [88].

#### 3.3.2. Solute Carrier Family 12 Member 8 (SLC12A8)

The solute carrier family 12-member 8 genes are located on chromosome 3 (3q21.2) and code for a sodium, potassium and chloride transporter related to control of keratinocyte proliferation. On this basis, it is directly implicated in developing psoriasis and is also known as *PSORS5* (psoriasis susceptibility 5) [89].

The *SLC12A8* rs651630 (G > A) polymorphism was evaluated in a study of predictive biomarkers for the risk of developing toxicity and/or paradoxical psoriasis due to anti-TNF drugs in Spanish patients with moderate-to-severe psoriasis (*n* = 161) [90]. The patients carrying the *SLC12A8* rs651630-AA genotype showed a higher risk of developing paradoxical psoriasis during treatment with anti-TNF medication (OR = 0, CI_95%_ = 0–0.06, *p* = 0.011) [90].

#### 3.3.3. Tumor Necrosis Factor Receptor Superfamily Member 1B (TNFRSF1B)

The TNF receptor superfamily member 1B (*TNFRSF1B*) is located on chromosome 1 (1p36.22) and encodes the TNF receptor responsible for recruiting the apoptotic suppressor proteins c-IAP1 and c-IAP2 [91]. This receptor mediates most of the metabolic effects of TNF, as it regulates the activity of this protein. Therefore, alterations in the *TNFRSF1B* gene may influence the TNF-mediated immune response, mainly in ETN, which inhibits the action of this receptor since ETN is the soluble p75 subunit of the TNF receptor [92].

The influence of the *TNFRSF1B* rs1061622 (T > G) polymorphism on response to anti-TNF drugs has been assessed in two studies and a meta-analysis [68,93,94]. The *TNFRSF1B* rs1061622-TT genotype has been associated with better response (PASI 75 at 6 months) in a study with 80 Greek patients (92.1% vs. 68%, *p* = 0.019), specifically to ETN treatment (100% vs. 60%; *p* = 0.001). However, no statistically significant association was found in treatment with INF (*n* = 22) and ADA (*n* = 14) [68]. The results were confirmed in a study with Spanish patients (*n* = 90) [93]. Patients carrying the *TNFRSF1B* rs1061622-G allele responded worse to anti-TNF drugs and to UTK (PASI 50 at 6 months) compared to those carrying the T allele (35% vs. 56%, *p* = 0.05), specifically patients treated with anti-TNF therapy (OR = 2.96, CI_95%_ = 1.09–8.02, *p* = 0.03) [93]. In addition, a meta-analysis evaluated the effect of this polymorphism on the response to anti-TNF medication in Asian and Caucasian patients with autoimmune pathologies, such as Crohn’s disease (7 studies/929 patients) [94]. The *TNFRSF1B* rs1061622-T allele was associated with better response to anti-TNF drugs (OR = 0.72, CI_95%_ = 0.57–0.93, *p* = 0.01). However, only two studies were conducted in patients diagnosed with moderate-to-severe psoriasis; the association was confirmed in this subgroup (*n* = 170) (OR = 0.39, CI_95%_ = 0.23–0.67, *p* < 0.001) [94].

#### 3.3.4. CD84 Molecule (CD84)

Located in the 1q23.3 region is the *CD84* gene, which encodes a membrane protein belonging to the signaling lymphocytic activation molecule (SLAM) family and to the CD2 subgroup of the immunoglobulin cell-surface receptor superfamily [95]. It is expressed in many types of immune cells, such as B and T cells regulating receptor-mediated signaling, and participates in the adhesion and activation of immune cells [95]. Alterations in this gene are, therefore, related to autoimmune pathologies.

The *CD84* rs6427528 (G > A) polymorphism alters the affinities of the transcription factor binding site in the 3′-UTR of *CD84*, leading to greater expression of the *CD84* gene in peripheral blood mononuclear cells, affecting the response to anti-TNF treatment [50]. This was demonstrated in a study in a population from the Netherlands (*n* = 161) where the *CD84* rs6427528-AG genotype was associated with better response (ΔPASI at 12 weeks) to ETN treatment (beta = −2.028, CI_95%_ = −3.794–0.261, *p* = 0.025) [50]. These results were confirmed in a meta-analysis of a genome-wide association study (GWAS) in Caucasian patients (13 studies/2706 patients) with rheumatoid arthritis treated with anti-TNF medication [96]. In particular, the *CD84* rs6427528-AG genotype was associated with greater effectiveness of ETN treatment in those patients (*n* = 733) (*p* = 0.004) [96].

#### 3.3.5. Fc Fragment of IgG Receptors IIA and IIIA (FCGR2A and FCGR3A)

Specific antibody receptors (FcR) are a group of surface receptors present in immune system cells (monocytes, macrophages, neutrophils, natural killer cells, and T and B lymphocytes), which interact with the constant or fragment crystallizable (Fc) region of antibodies. The receptors of the constant fraction of immunoglobulin G receptor gene family, in particular IIa (*FCGR2A*) and IIIa (*FCGR3A*), bind to the Fc region of immunoglobulin G (IgG), triggering a cell response of antibody-dependent cellular cytotoxicity (ADCC) mediated by phagocytic or cytotoxic cells [97].

The *FCGR2A* and *FCGR3A* genes are located on chromosome 1 (1q23.3), and alterations in these genes modify the affinity of the receptor for the immune complex [98]. The rs1801274 (A > C, G) polymorphism of the *FCGR2A* gene results in arginine (R) to histidine (H) substitution at position 131, reducing its affinity [99]. However, the *FCGR3A* rs396991 (A>C,G,T) polymorphism generates a change at position 158 from phenylalanine (F) to valine (V) [100]. It has been shown that the presence of the *FCGR3A*-V158 variant produces greater affinity for IgG, increasing the complement-mediated cytotoxic immune response and cell apoptosis, as well as the number of FcR receptors expressed on the membrane since patients carrying the *FCGR3A*-V158 variant express a larger number of these receptors on the surface of natural killer cells [101].

To date, several studies have been conducted evaluating the effect of these polymorphisms on the response to anti-TNF drugs, showing contradictory results [42,102,103,104,105]. The *FCGR3A*-158FF variant was associated with better response to anti-TNF medication at 6 weeks of treatment in a study with 35 American patients diagnosed with psoriatic pathology (*n* = 5) and rheumatoid arthritis (*n* = 30) (47.8% vs. 0%; *p* < 0.01) [102]. Subsequently, the impact of the *FCGR3A*-V158F and *FCGR2A*-H131R polymorphisms on anti-TNF response was assessed in a pilot study with biologic-naive Spanish patients diagnosed with moderate-to-severe psoriasis and treated with ADA, INF or ETN (*n* = 70) [103]. Both alleles were associated with a reduction in BSA at week 6 of treatment (beta = 0.42, *p* = 0.02 and beta = 0.425, *p* = 0.03 respectively), although these results were not confirmed at week 12 (*p* > 0.05) [103]. Batalla et al. corroborated the association between the *FCGR3A*-158FF polymorphism and anti-TNF response (PASI 75 at week 24) in a study with 115 biologic-naive Spanish patients diagnosed with moderate-to-severe psoriasis (OR = 12.05, CI_95%_ = 1.25–111.11, *p* = 0.04), although the association between the *FCGR2A*-131HH allele and anti-TNF response was not confirmed (*p* = 0.1) [104]. However, Mendrinou et al. evaluated the association between both polymorphisms and response to anti-TNF drugs (PASI 75 at 6 months) in 100 Greek patients diagnosed with moderate-to-severe psoriasis [105]. In particular, patients carrying the *FCGR3A*-158 V allele showed better anti-TNF response (OR = 2.96, CI_95%_ = 1.601–5.527, *p* = 0.018), especially to ETN, which achieved the best results (*n* = 55) (OR = 2.61, CI_95%_ = 1.078–6.402, *p* = 0.018), but no statistically significant association was found between the *FCGR2A*-H131R polymorphism and anti-TNF response (*p* = 0.882) [105]. Finally, a study in Spanish patients (*n* = 133) confirmed the association of the *FCGR2A*-H131R polymorphism with response (PASI 75 at week 6) to anti-TNF medication [42]. In particular, patients carrying the GG genotype showed a better response than those with the AG genotype (*p* = 0.015) [42].

#### 3.3.6. Interleukin 17 Receptor A (IL17RA)

The interleukin 17 receptors A (*IL17RA*) gene, located on chromosome 22 (22q11.1), encodes a membrane protein (IL17RA) which binds to interleukin 17A and 17 F and generates an inflammatory cascade [77]. This gene, therefore, plays a crucial role in developing psoriasis, being targeted by BDL [106,107].

Recent studies show an association between polymorphisms of *IL17RA* and response to treatment with biological therapy [80,81]. The rs4819554 polymorphism, located in the promoter region of *IL17RA*, was associated with response to anti-TNF drugs in Spanish patients (*n* = 238). Specifically, the *IL17RA* rs4819554-A allele was associated with better response (PASI 75 at 12 weeks) to anti-TNF treatment (OR = 1.86, CI_95%_ = 1.05–3.27, *p* = 0.03) [80].

#### 3.3.7. Interleukin 23 Receptor (IL23R)

The interleukin 23 receptor (IL-23R) is a subunit of the IL-23A/IL-23 receptor, which associates with IL-12RB1 to form the IL-23 receptor and induce T-cell, natural killer and macrophage stimulation by binding with IL-23 [108]. IL-23 is considered to be a proinflammatory cytokine, which participates in the acute response to infection in peripheral tissues [90]. The *IL23R* gene is located on chromosome 1 (1p31.3) and has demonstrated its importance in developing autoimmune inflammatory diseases and tumorigenesis [109,110].

The impact of polymorphisms in the *IL23R* gene on the response to anti-TNF drugs in biologic-naive patients diagnosed with moderate-to-severe psoriasis has been evaluated in a study with Spanish patients (*n* = 109). The *IL23R* rs11209026-GG genotype was associated with better treatment response at 6 months (PASI 90:66.3% vs. 0%, *p* = 0.006; ΔPASI: 86.8% vs. 67.8%, *p* = 0.013) [47]. Furthermore, the association between *IL23R* polymorphisms and the risk of developing toxicity and/or paradoxical psoriasis due to the anti-TNF medication has been demonstrated in a study with Spanish patients (*n* = 161) [90]. Specifically, the *IL23R* rs11209026-AG genotype was related to greater risk of developing paradoxical psoriasis during treatment (OR = 11,011.59, CI_95%_ = 17.36–6984187.8, *p* = 0.005) [90].

#### 3.3.8. Toll-Like Receptors

The Toll-like receptor (TLR) family consists of transmembrane proteins with an essential role in pathogen recognition and activation of the immune response. The external domain recognizes pathogen-associated molecular patterns (PAMPs) and damage-associated molecular patterns (DAMPs), while the TIR-type intracellular domain combines with IL-1, generating an inflammatory cascade.

##### Toll-Like Receptor 2 (TLR2)

The Toll-like receptor 2 (*TLR2*) gene is located on chromosome 4 (4q31.3). The function of the TLR2 protein is to recognize bacterial lipoproteins and other components of the microbial cell wall, cooperating with LY96 to mediate the innate immune response [111].

The influence of the *TLR2* rs4696480 (T > A) and rs11938228 (C > A, T) polymorphisms has been investigated in 478 patients from Denmark diagnosed with moderate-to-severe psoriasis and treated with anti-TNF medication (*n* = 376) and UTK (*n* = 230) [71]. The *TLR2* rs4696480-A allele (OR = 0.22, CI_95%_ = 0.08–0.59, *p* = 0.0032, q = 0.19) and the *TLR2* rs11938228-C allele (OR = 0.30, CI_95%_ = 0.14–0.64, *p* = 0.0019, q = 0.19) showed a worse response to anti-TNF treatment (PASI 75 at 3 months) [71]. However, no significant association was found in patients treated with UTK [71].

##### Lymphocyte Antigen 96 (LY96)

The lymphocyte antigen 96 (*LY96*) gene, also known as MD2 protein (*MD2*), is located on chromosome 8 (8q21.11) and encodes a protein, which cooperates with TLR2 and TLR4 in the immune response to the membrane lipopolysaccharides and cell-wall components of Gram-positive and Gram-negative bacteria [112].

Various genetic alterations of *LY96* have been studied. Specifically, the *LY96* rs11465996 (C > G) polymorphism has been evaluated in a cohort of patients from Denmark diagnosed with moderate-to-severe psoriasis and treated with anti-TNF drugs and UTK (*n* = 478), showing that patients carrying the *LY96* rs11465996-C allele had a worse response to UTK (ΔPASI at 3 months) (*n* = 230) (OR = 0.33, CI_95%_ = 0.15–0.71, *p* = 0.0044, q = 0.19) [71]. However, it was not possible to confirm this association in patients treated with anti-TNF medication [71].

##### TIR Domain-Containing Adapter Protein (TIRAP)

In region 11q24.2, we find the TIR domain-containing adapter protein (TIRAP) gene, also known as MYD88 adapter-like (*MAL*) [113]. *TIRAP* belongs to the group of genes containing a TIR domain and encodes a protein that interferes in the signaling cascade of TLR2 and TLR4 [114,115]. The possible genetic alterations of *TIRAP* could influence the immune response.

Previous studies have shown that the *TIRAP* rs8177374 (C > T; L180S) polymorphism interferes in the immune response by attenuating TLR2 signal transduction [113]. A study with 376 patients from Denmark diagnosed with moderate-to-severe psoriasis evaluated the effect of this polymorphism on response to UTK and anti-TNF drugs (PASI 75 at 12 weeks) [71]. In particular, patients carrying the *TIRAP* rs8177374-C allele showed a better response to UTK treatment (*n* = 230) compared to patients with the T allele (OR = 9.42, CI_95%_ = 1.96–45.3, *p* = 0.0051, q = 0.19) [71]. However, no statistically significant association was found between this polymorphism and anti-TNF response [71].

##### Toll-Like Receptor 5 (TLR5)

The Toll-like receptor 5 (TLR5) protein recognizes bacterial flagellins and recruits intracellular adapter proteins MYD88 and TRIF, leading to cytokine secretion and generating the inflammatory response. It, therefore, plays an important part in the relationship between the intestinal epithelium and enteric microbes and contributes to the composition of the intestinal microbiota throughout life. Its gene is located on chromosome 1 (1q41) [116,117].

Recently, the association of the *TLR5* rs5744174 (A > G) polymorphism with the response to biological drugs indicated in moderate-to-severe psoriasis (anti-TNF: *n* = 376; UTK: *n* = 230) has been evaluated in patients from Denmark [71]. Patients carrying the *TLR5* rs5744174-A allele showed a better response (ΔPASI at 3 months) to UTK treatment (OR = 5.26, CI_95%_ = 1.93–14.38, *p* = 0.0012, q = 0.19) [71]. However, it was not possible to confirm this association in patients treated with anti-TNF medication (*p* > 0.05) [71].

##### Toll-Like Receptor 9 *(TLR9)*

The Toll-like receptor 9 (*TLR9*) gene is located on chromosome 3 (3p21.2) and codes for a protein responsible for recognizing microbial nucleic acids (cytidine-phosphate-guanosine (CpG) dinucleotides) that induce the proliferation, activation, survival and production of B-cell antibodies [118].

The *TLR9* rs352139 (T > C, A, G) polymorphism has been associated with the response to treatment with biological therapy in patients diagnosed with psoriasis. A study conducted in 478 patients of Danish origin with moderate-to-severe psoriasis treated with anti-TNF drugs (*n* = 376) and/or UTK (*n* = 230) showed that patients carrying the *TLR9* rs352139-G showed greater effectiveness of anti-TNF treatment (drug survival at 225 days) (OR = 2.42, CI_95%_ = 1.32–4.44, *p* = 0.0044, q = 0.19) [71]. However, this association was not confirmed in patients treated with UTK [71].

#### 3.3.9. Peptidoglycan Recognition Protein 4 (PGLYRP4)

Peptidoglycan recognition protein 4 (PGLYRP4) is responsible for triggering the innate immune response and exercising bactericidal action on recognizing murein peptidoglycans of Gram-positive bacteria [119]. The *PGLYRP4* gene is located on chromosome 1 (1q21.3) and is directly implicated in the physiopathogenesis of psoriasis, as it plays an essential role in innate immunity; for this reason, it is also known as *PSORS4* (susceptibility to psoriasis 4) [120]. Therefore, possible genetic alterations could greatly influence the response to anti-TNF drugs or cytokine inhibitors.

A study assessed the impact of the *PGLYRP4–*24 rs2916205 (C > T) polymorphism on the response to anti-TNF drugs (PASI 75 at 3 and 6 months) in patients of Spanish origin diagnosed with psoriasis (*n* = 144) [42] and found an association between the *PGLYRP4–*24 rs2916205-C allele and worse anti-TNF response at 3 months of treatment (OR = 3.62, CI_95%_ = 1.00–13.07, *p* = 0.05) [42].

#### 3.3.10. F-Box and Leucine-Rich Repeat Protein 19 (FBXL19)

The F-box and leucine-rich repeat protein 19 (*FBXL19*) gene is situated in the 16p11.2 region and encodes a member of the Skp1-cullin-F-box family of E3 ubiquitin ligases. FBXL19 binds to the transmembrane receptor IL-1RL1 and regulates its ubiquitination and degradation, activating the innate immune system and MHC class I [121]. This gene has been linked to the regulation of pulmonary inflammation and psoriasis. Therefore, alterations of the *FBXL19* gene may influence the response to biologics indicated in moderate-to-severe psoriasis.

The *FBXL19* rs10782001 (G > A, C) polymorphism was evaluated in a study of predictive biomarkers for the risk of developing toxicity and/or paradoxical psoriasis due to anti-TNF drugs in Spanish patients with moderate-to-severe psoriasis (*n* = 161) [87]. Patients carrying the *FBXL19* rs10782001-GG genotype showed a higher risk of developing paradoxical psoriasis during treatment (OR = 32.85, CI_95%_ = 1.46–738.37, *p* = 0.0028) [90].

### 3.4. Other Genes

In recent years, studies have been conducted on other genes implicated in the pathogenesis of psoriasis that may exercise a crucial role in response to treatment with biological therapies in patients diagnosed with psoriatic disease [122].

The protein encoded by the *CDKAL1* gene, located on chromosome 6 (6p22.3), belongs to the methylthiotransferase family and catalyzes the methylthiolation of N6-threonylcarbamoyladenosine, producing 2-methylthio-N6-threonylcarbamoyladenosine at position 37 in the transfer RNA that reads codons beginning with adenine (AAA and AAG) [123]. This gene has been associated with autoimmune pathologies such as non-insulin-dependent diabetes mellitus [124]. There are various genetic alterations of *CDKAL1*; specifically, the rs6908425 (C > A, T) variant affects the response to anti-TNF drugs, as has been demonstrated in two studies [42,125]. Coto-Segura et al., associated the *CDKAL1* rs6908425-C allele with better response (PASI 75 at 24 weeks) in 116 Spanish patients diagnosed with moderate-to-severe psoriasis (OR = 3.14, CI_95%_ = 1.40–7.05, *p* = 0.005) [125]. Conversely, these results were not validated in a cohort of Spanish patients (*n* = 133) since patients carrying the *CDKAL1* rs6908425-C allele showed worse treatment response at six months (*p* = 0.013) [42].

The caspase recruitment domain family member 14 (*CARD14*) gene, which has a mutation named *PSORS2* because of its relationship to psoriasis, encodes a scaffolding protein belonging to the membrane-associated guanylate kinase (MAGUK) family of proteins involved in various cellular processes, such as cellular adhesion, signal transduction and control of cell polarity. It has been shown that this protein interacts specifically with BCL10, a protein that positively regulates cell apoptosis and activates NF-kβ [126]. Genetic alterations may influence the response to biologics indicated for treating psoriasis. The *CARD14* rs11652075 (C > T; R820T) polymorphism produces a change in position 820 from tryptophan (T) to arginine (R), and its usefulness as a predictive biomarker of response to anti-TNF drugs (PASI 75 at week 24) has been evaluated in Spanish patients (*n* = 116). The results of the study revealed that the patients carrying the *CARD14* rs11652075-CC genotype showed better response compared to those with the T allele (OR = 3.71, CI_95%_ = 1.30–10.51, *p* = 0.01) [127].

On the other hand, a recent study analyzed 124 polymorphisms of a number of candidate genes associated with response to ETN (PASI 75 at 3 and 6 months) in 78 Spanish patients diagnosed with moderate-to-severe plaque psoriasis; it found that the *PTTG1* rs2431697 (T > C), *MAP3K1* rs96844 (G > A) and *ZNF816A* rs9304742 (T > C) polymorphisms were associated with good response to ETN at 3 months of treatment (*PTTG1* rs2431697-T: *p* = 0.04; *MAP3K1* rs96844-C: *p* = 0.009; *ZNF816A* rs9304742-T: *p* = 0.006). In addition, the G allele of the *GBP6* rs928655 (G > A, C) polymorphism has been associated with better response to ETN at 6 months (*n* = 68; *p* = 0.014) [40]. The association of the *MAP3K1* rs96844 and *ZNF816A* rs9304742 polymorphisms with good response to anti-TNF medication (PASI 75 at 3 and 6 months) was subsequently confirmed in a study with 144 Spanish patients diagnosed with psoriasis (*MAP3K1* rs96844-C at 3 months (*p* = 0.004) and at six months (*p* = 0.045) and *ZNF816A* rs9304742-T at 3 months (*p* = 0.02)).

Two further polymorphisms associated with anti-TNF response (PASI 75 at 3 and 6 months) were also identified. In particular, the A allele of the *CTNNA2* rs11126740 (A > C, G, T) polymorphism showed worse response at 3 months (*n* = 144; *p* = 0.003) and patients carrying the T allele of the *HTR2A* rs6311 (C > A, T) polymorphism were associated with worse response at 6 months (*n* = 133; *p* = 0.038) [42].

Finally, it is worth highlighting the only study that has evaluated the influence of the presence of particular genetic polymorphisms on susceptibility to developing toxicity and/or paradoxical psoriasis due to anti-TNF drugs, conducted in 161 Spanish patients diagnosed with plaque psoriasis (Table 3) [90]. Specifically, it showed that patients carrying the *CTLA4* rs3087243-AG/GG or *TAP1* rs1800453-AG genotypes, or the previously mentioned *FBXL19* rs10782001-GG, *IL23R* rs11209026-AG and *SLC12A8* rs651630-AA, had a higher risk of developing paradoxical psoriasis during treatment (*CTLA4* rs3087243-AG/GG: OR = 0.001, CI_95%_ = 0–0.24, *p* = 0.012; *TAP1* rs1800453-AG: OR = 0.009, CI_95%_ = 0–0.45, *p* = 0.018) [90]. There are few statistically significant results between genetic polymorphisms and developing toxicity in patients diagnosed with psoriasis treated with biological therapies. However, in other therapies, such as methotrexate, an association has been found. Particularly, patients carriers of the Betaine-homocysteine S-methyltransferase (BHMT) rs3733890 variant allele have showed an increased risk of hepatotoxicity (OR = 3.17, CI95% = 1.18–8.49, *p* = 0.022) [122]. 

## 4. Conclusions

There is a wide range of biologics indicated for moderate-to-severe psoriasis that have proved effective and safe; however, certain patients do not obtain the expected effect in the short or long term and experience various degrees of toxicity. This variability in short- and long-term response and in toxicity may be due to genetic factors. Precision medicine research has assessed the influence of polymorphisms of genes involved in the pathological environment of the disease, metabolism or mechanism of action on the efficacy of these drugs. Specifically, the allelic variants of HLA genes have been extensively studied, but the results are contradictory. The *HLA-A* rs9260313/*TRAF3IP2* rs13190932 and HLA-Cw*06/LCE3C_LCE3B del/*ins* haplotypes, together with the *HLA-B/MICA* rs13437088 and *HLA-C* rs12191877, rs1048554 and rs610604 polymorphisms, have shown an association with the response to anti-TNF drugs. However, the presence of the HLA-Cw*06 alleles has been shown to be associated with response to UTK but not to anti-TNF drugs. In addition, associations with response to anti-TNF drugs in patients diagnosed with moderate-to-severe psoriasis have been found for polymorphisms of the following genes: *TNF* (*TNF*-238 rs361525, *TNF*-308 rs1800629, *TNF*-857 rs1799724, *TNF*-1031 rs1799964), *IL6* (rs1800795), *IL12B* (rs2546890), *TNFAIP3* (rs6920220), *PDE3A-SLCO1C1* (rs11045392-rs3794271), *TNFRSF1A* (rs1061622), *CD84* (rs6427528), *FCGR2A* (rs1801274), *FCGR3A* (rs396991), *IL17RA* (rs4819554), *IL23R* (rs11209026), *TLR2* (rs4696480 and rs11938228), *TLR9* (rs352139), *PGLYRP4–*24 (rs2916205), *CDKAL1* (rs6908425), *CARD14* (rs11652075), *PTTG1* (rs2431697), *MAP3K1* (rs96844), *ZNF816A* (rs9304742), *GBP6* (rs928655), *CTNNA2* (rs11126740) and *HTR2A* (rs6311). Furthermore, polymorphisms of the following genes have shown an association with response to UTK: *IL12B* (rs3213094), *TNFAIP3* (rs610604), *LY96* (rs11465996), *TIRAP* (rs8177374) and *TLR5* (rs5744174), and the *IL1B* rs1143623 and rs1143627 and *IL17F* rs763780 polymorphisms have proved to be associated with susceptibility to developing toxicity and paradoxical psoriasis due to anti-TNF drugs.

In general, we have summarized the genetic polymorphisms that have been shown to play a significant role in the effectiveness of biopharmaceuticals in moderate-to-severe psoriasis. However, this review has several limitations. First, many of the existing studies have small sample sizes, which makes it difficult to reach a definitive interpretation of the role of these genes in response to treatment with biopharmaceuticals. Second, the studies show a lack of uniformity in their definitions of the treatment response and follow-up period, which impacts the possibility of generalizing from the results. Third, most of the studies have analyzed the response to anti-TNF drugs as a group, despite the fact that they have different molecular structures and degrees of efficacy, giving rise to the opacity of results. Finally, new targets have been introduced in the treatment algorithm for moderate-to-severe psoriasis, which has proved more effective than drugs than inhibit TNF or IL-12, IL-17 and IL-23. However, pharmacogenetic markers for these drugs are not yet available. Consequently, new studies are needed to look for specific biomarkers for each drug with a uniform definition of response, longer-term follow-up and larger sample sizes to confirm these results.

In conclusion, the results suggest that polymorphisms detected in the HLA genes, in genes that encode cytokines (*TNF*, IL genes, *TNFAIP3*), transporters (*PDE3A*-*SLCO1C1*, *SLC12A8*), receptors (*TNFRSF1B*, *CD84*, *FCGR2A and FCGR3A*, *IL17RA*, *IL23R*, TLR genes, *PGLYRP4*) and associated proteins (*TNFAIP3*, *LY96*, *TIRAP*, *FBXL19*), as well as other genes implicated in the pathogenesis of psoriasis (*CDKAL1*, *CARD14*, *PTTG1*, *MAP3K1*, *ZNF816A*, *GBP6*, *CTNNA2*, *HTR2A*, *CTLA4*, *TAP1*) can be used in the future as predictive markers of treatment response and/or toxicity with biological therapies in patients diagnosed with moderate-to-severe psoriasis, tailoring treatment to the individual patient.

## Figures and Tables

**Table 1:** Human leukocyte antigens (HLA) haplotypes and polymorphisms investigated in response to biological therapies in psoriasis patients.

Gene	SNP	Year	N	Population	Drugs	Response		Results			Responsive Allele or Genotype	PMID
						Time (Months)	Outcome Measure	OR	CI_95%_	*p*-value		
Haplotype HLA-A/ TRAF3IP2	rs9260313/ rs13190932	2018	109	Spain	INF ADA	6	PASI75	-	-	<0.05 ^a^	GT	28921458
Haplotype HLA-B	HLA-B*46	2012	74	China	ETN	3	PASI50	-	-	1 ^b^	-	21985130
					UTK					0.32 ^b^		
HLA-B/MICA	rs13437088	2017	81	Spain	ETN	3	PASI75	589.99	2.71–128,614.40	0.02	TT	28470127
HLA-C	rs12191877	2016	144	Spain	Anti-TNF	3	PASI75	0.30	0.11–0.88	0.027	T	27670765
	rs1048554	2016	250	Greece	Anti-TNF	6	PASI75	3.94	1.16–13.3	0.0032	C	27043841
	rs610604				ADA	6	PASI75	-	-	0.05 ^b^	A	
	-	2013	109	Spain	Anti-TNF	6	PASI75	85.1	71.7–93.8	0.049	(-)	23662788
	-	2013	123	Italy	ETN INF	3	PASI75	-	-	>0.05	(+)	-
	-	2014	138	United Kingdom and Ireland	ADA ETN	6	PASI75	-	-	>0.05	(+)	24758522
	HLA-Cw*06/ LCE3C_ LCE3B del/ins	2015	116	Spain	Anti-TNF	3	PASI75	3.14	1.07–9.24	0.034	(+)/del	25794162
	-	2016	96	Italy	ETN	3	PASI75	-	-	>0.05	(+)	27348478
	-	2017	122	Italy	ADA	3	PASI75	1.11	0.52–2.36	0.78	(+)	28130758
	-	2013	51	Italy	UTK	1	PASI75	5.36	1.24–23.1	0.024	(+)	23521149
						3	PASI75	13.4	1.6–12.6	<0.008		
						3	PASI90	4.6	-	0.02		
						10	PASI75	3.9	2–7.37	0.014		
						10	PASI90	8.7	-	0.012		
	-	2016	134	Italy	UTK	3	PASI75	4.1	-	0.001	(+)	26775778
						13	PASI75	3.7	-	0.003		
	-	2017	255	Belgium, Italy, Netherlands	UTK	3	PASI75	3.28	1.92–5.59	<0.001	(+)	28207934
						13	PASI75	3.82	1.88–7.73	<0.001		
	-	2014	66	China	UTK	4	PASI75	0.28	0.11–0.68	0.005	(+)	24734995
	-	2016	332	United States	UTK	3	PASI75	-	-	<0.05 ^b^	(+)	27476722
HLA-C*06:02 (HLA-Cw*06	HLA-Cw6/L12B rs3212227	2016	64	Italy	UTK	1	PASI75	10.49	50–0	0.009	(+)/C	26678060
						13		5.21	83.3–44.4	0.007		
	HLA-Cw6/IL12B rs6887695					1	PASI75	6.11	23.5–10.5	0.031	(+)/GG	
						13		4.75	82.4–42.1	0.006		
	HLA-Cw6 y IL6 rs1800795					1	PASI75	6.52	38.5–0	0.027	(+)/C	
						13		4.99	84.9–46.7	0.005		
	-	2019	1048	Caucasians and Asian	UTK	6	PASI75	0.24	0.14–0.35	<0.001	(+)	30994858
	-	2019	1326	United Kingdom and Ireland	ADA	6	PASI90	2.95	-	<0.001	(-)	30578879
HLA-G 14-pb ins/del	rs66554220	2014	11	Italy	Anti-TNF	4	PASI75	-	-	0.7 ^b^	(+)	24909182

N: number of patients; OR: odds ratio; CI_95%:_ 95% confidence interval; ADA: adalimumab; ETN: etanercept; INF: infliximab; UTK: ustekinumab; anti-TNF: inhibitors TNF drugs; PASI: psoriasis area and severity index; PASI75:75% improvement from baseline PASI; PASI90:90% improvement from baseline PASI; (+) presence of allele HLA-C*06:02; (-) absence of allele HLA-C*06:02; ^a^ *p*-value for chi-squared test; ^b^ *p*-value for Fisher’s test. The script means that the paper did not provide any information on this parameter.

**Table 2:** Gene polymorphisms involved in response to biological therapies.

Gene	SNP	Year	N	Population	Pathology	Drugs	Response		Results			Responsive Allele or Genotype	PMID
							Time (Months)	Outcome Measure	OR	CI_95%_	*p*-value		
TNF-α-238	rs361525	2013	109	Spain	PS	Anti-TNF	6	PASI75	-	-	0.049 ^f^	G	23662788
TNF-α-857	rs1799724						-	BSA	-	-	0.004 ^f^	C	
							-	PASI	-	-	0.009 ^f^		
							6	PASI75	-	-	0.006 ^f^		
TNF-α-1031	rs1799964						3	PASI75	-	-	0.047 ^f^	TT	
							6				0.038 ^f^		
TNF-α-308	rs1800629	2015	807	Caucasians	AE IBD APS PS	Anti-TNF	-	-	2.005	1.417–2.838	0.000086	G	26244882
TNF-α-238	rs361525		500						2.196	1161–4.154	0.016	G	
TNF-α-857	rs1799724		483						1.779	1.13–2.802	0.013	C	
			177		PS	ETN	-	-	2.238	1.319–3.798	0.003	C	
IL1-β	rs1143623	2017	376	Denmark	PS	Anti-TNF	3	PASI75	0.35	-	0.0041	GG	28696418
			230			UTK			0.25	-	0.0049		
	rs1143627	2017	376		PS	Anti-TNF	3	PASI75	0.28	-	0.0016	AA	
			230			UTK			0.24	-	0.0042		
IL6	rs1800795	2012	60	Italy	PS	Anti-TNF	6	PASI75	2.00	1.19–3.38	≤0.05	GG	22158445
IL12β	rs2546890	2017	78	Spain	PS APS	ETN	6	PASI75	11.92	1.07–132.67	0.044	G	28470127
		2017	144	Spain	PS	Anti-TNF	3	PASI75	3.22	1.23–8.40	0.017	G	27670765
							6		4.14	1.23–13.81	0.022		
							12		2.79	1.02–7.64	0.046		
	rs3213094	2017	66	Netherlands	PS	UTK	3	ΔPASI	−3.15 ^c^	−5.724– −0.586	0.017	CT	27564082
IL17F	rs763780	2015	67	Spain	PS	UTK	4	PASI75	12.23	1.17–127.36	0.022	CT	26347322
							7		14.18	1.35–149.42	0.016		
			62			ADA	7	PASI75	14.00	2.15–91.12	0.0044		
TNFAIP3	rs610604	2013	51	Italy	PS	UTK	10	PASI75	1.6	-	0.75	-	23521149
		2016	64	Italy	PS	UTK	3	PASI75	-	-	>0.05	-	26678060
		2017	66	Netherlands	PS	UTK	3	ΔPASI	3.490 ^c^	0.329–6.650	0.031	GG	27564082
					PS APS				11.230 ^c^	7.486–14.973	<0.001		
		2019	20	Spain	PS APS	Anti-TNF	3	% EQ-VAS	−10.60	−20.71–0.048	0.041	AC/CC	30653751
	rs6920220								−25.83	−47.969– −3.698	0.025	AA	
PDE3A-SLCO1C1	rs11045392-rs3794271	2015	130	Spain	PS	Anti-TNF	3	ΔPASI	-	-	0.00057	G	25403996
TNFRSF1B	rs1061622	2012	80	Greece	PS	Anti-TNF	6	PASI75	-	-	0.019 ^f^	TT	22111980
						ETN			-	-	0.001 ^f^		
		2015	90	Spain	PS	Anti-TNF	6	PASI50	2.96	1.09–8.02	0.03	G	25537528
		2015	929	Caucasians and Asian	RA CD PS	Anti-TNF	-	-	0.72	0.57–0.93	0.01	T	26071216
		2015	170		PS		-	-	0.39	0.23–0.67	<0.001	T	
CD84	rs6427528	2013	733	Caucasians	RA	ETN	-	-	-	-	0.004	AG	23555300
		2017	161	Netherlands	PS	ETN	3	ΔPASI	−2.028 ^c^	−3.794–0.261	0.025	AG	27564082
FCGR2A	rs1801274	2013	70	Spain	PS	Anti-TNF	6^a^	dBSA	-	-	0.03 ^d^	131HH	24048425
		2015	115	Spain	PS	Anti-TNF	6	PASI75	-	-	0.1 ^e^	-	26398016
		2016	100	Greece	PS	Anti-TNF	6	PASI75	-	-	0.882 ^e^	H131R	27044681
		2016	133	Spain	PS	Anti-TNF	6^a^	PASI75	13.32	1.67–106.50	0.015	131RR	27670765
FCGR3A	rs396991	2005	35	United States	PS RA	Anti-TNF	6^a^	-	-	-	<0.01 ^f^	158FF	16142749
		2013	70	Spain	PS	Anti-TNF	6^a^	dBSA	-	-	0.02 ^d^	158FF	24048425
		2015	115	Spain	PS	Anti-TNF	6	PASI75	12.05	1.25–111.11	0.04	158FF	26398016
		2016	100	Greece	PS	Anti-TNF	6	PASI75	2.96	1.601–5.527	0.0018	158 V	27044681
IL17RA	rs4819554	2018	238	Spain	PS	Anti-TNF	3	PASI75	1.86	1.05–3.27	0.03	A	27670766
IL23R	rs11209026	2013	109	Spain	PS	Anti-TNF	6	PASI90	-	-	0.006^f^	GG	23662788
TLR2	rs4696480	2017	376	Denmark	PS	Anti-TNF	3	PASI75	0.22	0.08–0.59	0.0032	A	28696418
	rs11938228								0.30	0.14–0.64	0.0019	C	
LY96	rs11465996		230			UTK	3	ΔPASI	0.33	0.15–0.71	0.0044	C	
TIRAP	rs8177374		230				3	PASI75	9.42	1.96–45.3	0.0051	C	
TLR5	rs5744174						3	ΔPASI	5.26	1.93–14.38	0.0012	A	
TLR9	rs352139		376			Anti-TNF	225^b^	DS	2.42	1.32–4.44	0.0044	G	
PGLYR4-24	rs2916205	2016	144	Spain	PS	Anti-TNF	3	PASI75	3.62	1.00–13.07	0.05	C	27670765
CDKAL1	rs6908425	2015	116	Spain	PS	Anti-TNF	6	PASI75	3.14	1.40–7.05	0.005	CC	26563541
		2016	133	Spain	PS	Anti-TNF	6	PASI75	0.14	0.03–0.66	0.013	T	27670765
CARD14	rs11652075	2016	116	Spain	PS	Anti-TNF	6	PASI75	3.71	1.30–10.51	0.01	CC	26854129
PTTG1	rs2431697	2017	78	Spain	PS APS	ETN	3	PASI75	29.80	1.16–765.68	0.04	C	28470127
MAP3K1	rs96844	2017	78	Spain	PS APS	ETN	3	PASI75	0.01	0–0.33	0.009	C	28470127
		2016	144	Spain	PS	Anti-TNF	3	PASI75	0.17	0.05–0.56	0.017	C	27670765
							6	PASI75	0.24	0.06–0.97	0.045		
ZNF816A	rs9304742	2017	78	Spain	PS APS	ETN	3	PASI75	8144.11	13.03–5089337.0	0.006	CC	28470127
		2016	144	Spain	PS	Anti-TNF	3	PASI75	7.66	1.37–42.70	0.02	CC	27670765
GBP6	rs928655	2017	68	Spain	PS APS	ETN	6	PASI75	0.14	0.03–0.67	0.014	G	28470127
CTNNA2	rs11126740	2016	144	Spain	PS	Anti-TNF	3	PASI75	20.56	2.75–153.69	0.003	AA	27670765
HTR2A	rs6311						6	PASI75	5.6	1.10–28.63	0.038	T	

N: number of patients; OR: odds ratio; CI_95%_: 95% confidence interval; ADA: adalimumab; ETN: etanercept; UTK: Ustekinumab; anti-TNF-α: inhibitors TNF-α drugs;% EQVAS: European quality of life visual analog scale; PASI: psoriasis area and severity index; PASI75:75% improvement from baseline PASI; PASI90:90% improvement from baseline PASI; dBSA: decrease of BSA; DS: drug survival; APS: psoriatic arthritis; CD: Crohn’s disease; EA: ankylosing spondylitis; IBD: inflammatory bowel disease; PS: psoriasis; RA: rheumatoid arthritis; ^a^ weeks; ^b^ days; ^c^ beta; ^d^ *p*-value adjusted by age, sex, initial BSA, and the efficacy of the anti-tumor necrosis factor agent (percentage of PASI improvement) in week 6; ^e^ *p*-value for the chi-squared test; ^f^ *p*-value for Fisher’s test. The script means that the paper did not provide any information on this parameter.

**Table 3:** Gene polymorphisms involved in toxicity to anti-TNF-α therapies in psoriasis patients.

Gen	SNP	Year	N	Results			Responsive Allele or Genotype	PMID
				OR	CI_95%_	*p*-value		
CTLA4	rs3087243	2016	161	0.001	0–0.24	0.012	AG/GG	26194362
FBXL19	rs10782001			32.85	1.46–738.37	0.0028	GG	
IL23R	rs11209026			11,011.59	17.36–6984187.8	0.005	AG	
SLC12A8	rs651630			0	0–0.06	0.011	AA	
TAP1	rs1800453			0.009	0–0.45	0.018	AG	

N: number of patients; OR: odds ratio; CI_95%_: 95% confidence interval.
